# Vitamin A Impairs the Reprogramming of Tregs into IL-17-Producing Cells during Intestinal Inflammation

**DOI:** 10.1155/2015/137893

**Published:** 2015-10-25

**Authors:** Gabriela Tejón, Valeria Manríquez, Jaime De Calisto, Felipe Flores-Santibáñez, Yessia Hidalgo, Natalia Crisóstomo, Dominique Fernández, Daniela Sauma, J. Rodrigo Mora, María R. Bono, Mario Rosemblatt

**Affiliations:** ^1^Departamento de Biología, Facultad de Ciencias, Universidad de Chile, 7800024 Santiago, Chile; ^2^Gastrointestinal Unit, Department of Medicine, Massachusetts General Hospital, Harvard Medical School, Boston, MA 02114, USA; ^3^Fundación Ciencia & Vida, 7780272 Santiago, Chile; ^4^Facultad de Ciencias Biológicas, Universidad Andres Bello, 8370146 Santiago, Chile

## Abstract

Maintaining the identity of Foxp3^+^ regulatory T cells (Tregs) is critical for controlling immune responses in the gut, where an imbalance between Tregs and T effector cells has been linked to inflammatory bowel disease. Accumulating evidence suggests that Tregs can convert into Th17 cells and acquire an inflammatory phenotype. In this study, we used an adoptive transfer model of Ag-specific T cells to study the contribution of different factors to the reprogramming of *in vitro*-generated Treg cells (iTreg) into IL-17-producing cells in a mouse model of gut inflammation *in vivo*. Our results show that intestinal inflammation induces the reprogramming of iTreg cells into IL-17-producing cells and that vitamin A restrains reprogramming in the gut. We also demonstrate that the presence of IL-2 during the *in vitro* generation of iTreg cells confers resistance to Th17 conversion but that IL-2 and retinoic acid (RA) cooperate to maintain Foxp3 expression following stimulation under Th17-polarizing conditions. Additionally, although IL-2 and RA differentially regulate the expression of different Treg cell suppressive markers, Treg cells generated under different polarizing conditions present similar suppressive capacity.

## 1. Introduction

Despite many years of investigation, the etiology of inflammatory bowel diseases (IBDs) such as ulcerative colitis or Crohn's disease remains unknown. Present day hypotheses propose that abnormal immune responses in the gut mucosa cause continual intestinal inflammation, specifically alterations in the T cell response either to the gut flora or to normal components of the gut lumen [1, 2]. A number of animal models have helped shed light on the possible mechanisms involved in the immunological imbalance found in these diseases [3].

Some reports have implicated CD4^+^ T cells in both the initiation and maintenance of chronic inflammation of the gut. An imbalance in the development and function of IL-17-producing Th17 cells and Foxp3^+^ Treg in the intestine plays an important role in IBD [4, 5]. Moreover, several studies have described that Th17-related cytokines such as IL-17 and Th1 cytokines (tumor necrosis factor, IL-12, and interferon-*γ*) are noticeably augmented in the inflamed gut [6–8]. Although the exact role of Th17 cells in the pathogenesis of intestinal bowel diseases is not well understood, recent studies evaluating the role of Th1 and Th17 cells in the induction of experimental colitis in mice have shown a prevalence of Th17 cells over their Th1 counterpart in the inflamed gut [9].

A number of recent reports using different animal models have demonstrated the importance of gut-resident Treg cells in preventing the development of experimental colitis [10–12]. More interestingly, studies have shown that the transfer of Treg cells into mice undergoing experimental colitis can resolve an already established disease [13, 14], which indicates that manipulating the T cell compartment may help in the resolution of chronic inflammatory diseases.

Additionally, all-trans-retinoic acid (RA), a vitamin A metabolite that is produced by specialized dendritic cells (DCs) in the gut, modulates Foxp3^+^ regulatory T cell and Th17 effector T cell differentiation [15]. Several* in vitro* studies have demonstrated that RA enhances the TGF-*β*-induced differentiation of naive CD4^+^ T cells into Treg cells [16–18] while simultaneously inhibiting proinflammatory Th17 differentiation [19]. However, the* in vivo* effects of RA on Treg/Th17 modulation have not been fully elucidated, and the experimental results appear to vary depending on the system used [19–21].

TGF-*β* is a pleiotropic cytokine that is involved in the generation of both Th17 and Treg cells, depending on the other cytokines present in the local environment. Thus, T cells activated with IL-2 and TGF-*β* become Foxp3^+^ regulatory T cells [22], whereas activation with IL-6 and IL-1*β* results in Th17 cells [23]. Moreover, accumulating evidence suggests that Tregs cells can lose Foxp3 expression and be reprogrammed to express IL-17 under certain circumstances. Using a model of lymphopenic mice, Yurchenko and colleagues reported that nTreg from the thymus and peripheral lymphoid organs can be reprogrammed to Th17 (and to Th1) cells in the gut [24]. Additionally, using a mouse model of rheumatoid arthritis, the Komatsu group described that Th17 cells with autoimmune properties can be generated from Foxp3^+^ regulatory T cells* in vivo*, demonstrating that the instability of Treg can lead to pathological conditions [25]. Thus, defining the mechanisms and factors controlling Treg cell plasticity may contribute to our understanding of the immune deregulation observed in chronic inflammation and autoimmunity and may be helpful when designing regulatory T cell-based therapies.

In the current study, we demonstrate that the conversion of Foxp3^+^ regulatory T cells into IL-17 producer cells can be regulated by inflammatory signals in the gut. We further report that IL-2 and RA confer Treg cells with resistance against converting to Th17 cells.

## 2. Materials and Methods

### 2.1. Mice

C57BL/6 and OTII mice were purchased from Jackson Laboratories and housed under specific pathogen-free conditions. Foxp3-GFP (CD45.1^+^) knock-in mice were generously provided by Dr. Alexander Rudensky, Howard Hughes Medical Institute, Memorial Sloan-Kettering Cancer Center, New York. OTII/Foxp3-GFP (CD45.1^+^/CD45.2^+^) mice were generated by crossing OT-II mice (CD45.2^+^) with Foxp3-GFP knock-in mice (CD45.1^+^). Vitamin A-deficient (VAD) mice were obtained by feeding mice with a vitamin A-deficient diet from day 10 of gestation.

Animal work was carried out under institutional regulations of the Fundación Ciencia & Vida and the Faculty of Sciences, Universidad de Chile. All animal work was approved by and performed within the guidelines of the local Animal Care and Use Committee.

### 2.2. Antibodies and Reagents

The following antibodies from eBioscience (San Diego, CA, USA) were used: anti-mouse CD4 APC-H7 (GK1.5), CD25 PE (PC61.5), IL-17 PE (eBio17B7), IL-17 APC (eBio17B7), Foxp3 PE-Cy7 (FJK-16s), IgG 2a PE-Cy7 (eBR2a), CD39 PE (24DMS1), CD73 PE-Cy7 (Ty/11.8), CTLA-4 PE (UC10-4F10-11), Lag-3 APC (C9b7w), CCR9 PE (eBioCW-1.2), *α*4*β*7 PE (DATK32), B220 (RA3-6b2), CD19 APCH-7 (1D3), CD11c (N418), CD62L FITC (MEL-14), CD45.1 PE-cy7 (A20) purified anti-CD3 (145-2C11), and purified CD16/32 (93). From Biolegend (San Diego, CA, USA), we used CD4 PECy7 (GK 1.5), I-Ab FITC (25-9-17) and CD11b APC (M1/70), and purified *α*-IFN*γ* (XMG1.2). Recombinant mouse IL-2, TGF-*β*1, IL-6, and IL-1*β* were purchased from eBioscience. All-trans-retinoic acid, OVA protein, PMA, and ionomycin were purchased from Sigma Aldrich (St. Louis, MO, USA).

### 2.3. Isolation of OVA-Specific Naive T Cells

Splenic CD4^+^ cells from OTII/Foxp3-GFP mice were enriched using the Miltenyi CD4^+^ T cell isolation kit II (Miltenyi Biotech, Bergisch-Gladbach, Germany) according to the manufacturer's instructions. Naive CD4^+^CD25^−^ CD62L-CD44int T cells were further purified by cell sorting using FACS ARIA II (Becton Dickinson, NJ, USA) after surface staining with specific anti-mouse antibodies.

### 2.4. Isolation of Splenic APCs

Spleen tissue was fragmented and digested for 45 min at 37°C in the presence of collagenase D (Roche, Mannheim, Germany) and 2 *μ*g/mL of DNAse I (Roche) in PBS plus 10% fetal bovine serum. Undigested fibrous material was removed by filtration through a cell strainer. CD11c^+^ cells were obtained by positive selection using anti-CD11c microbeads (Miltenyi Biotec) according to the manufacturer's instructions. The cells were assayed for the presence of DCs and B cells by flow cytometry, obtaining an average of 60% CD11c^+^ DCs and 40% B220^+^ B cells.

### 2.5. *In Vitro* T Cell Differentiation and Reprogramming

To generate Treg cells, naive CD4^+^ T cells were cocultured with APC cells at a 5 : 1 ratio in the presence of 1 *μ*g/mL anti-CD3, under iTreg polarizing conditions (5 ng/mL TGF-*β*, 5 ng/mL IL-2, and 10 nM retinoic acid). iTreg (CD4^+^Foxp3^+^) cells were purified by cell sorting and cocultured with dendritic cells at a 5 : 1 ratio in the presence of 1 *μ*g/mL anti-CD3, under Th17-polarizing conditions (5 ng/mL TGF-*β*, 20 ng/mL IL-6, 10 ng/mL IL-1*β*, and 10 *μ*g/mL anti-IFN-*γ*) for 6 days. To assess IL-17 production, T cells were activated for 4 h with PMA plus ionomycin in the presence of BFA. The cells were fixed and permeabilized with fixation/permeabilization working solution (BD Bioscience) according to the manufacturer's instructions.

### 2.6. T Cell Suppression Assay

For the Treg suppression assays, CD4^+^CD25^−^ responder T cells (0.1 × 10^6^) from C57BL/6 mice were labeled with CellTrace Violet (Invitrogen) and stimulated with (2 × 10^4^) C57BL/6 APCs plus 1 *μ*g/mL anti-CD3 mAb. iTregs purified by sorting were added at different ratios. After 3 days, CellTrace Violet dilution in the responder cells was analyzed by flow cytometry.

### 2.7. Adoptive Transfer of iTregs for* In Vivo* Stability Assays

*In vitro*-generated iTregs from OT-II/Foxp3-GFP mice (CD4^+^GFP^+^CD45.1/2^+^, 1 × 10^6^) were sorted (FACS Aria II) and intravenously transferred to C57BL/6 (CD45.2^+^) mice. At days 1 and 3 after the adoptive transfer, the recipient mice were injected intraperitoneally with either 1 mg OVA alone or OVA plus a CD3-specific antibody (clone 2C11, 10 mg per mouse). Six days later, lymphoid organs and lamina propria were dissected as previously described [26], and the cells were analyzed by flow cytometry to assess the expression of Foxp3-GFP and IL-17 production on the transferred cells (CD45.1^+^).

### 2.8. Statistical Analyses

The data are presented as the means ± SEM and were analyzed using GraphPad Prism Software 5.0c (La Jolla, CA). Significance was determined using the Mann-Whitney *U* test or repeated measures ANOVA with Bonferroni's posttest. Significance was set at *p* < 0.05.

## 3. Results

### 3.1. Vitamin A Impairs the Reprogramming of Treg Cells into IL-17-Producing Cells during Acute Intestinal Inflammation* In Vivo*

**Figure 1:**
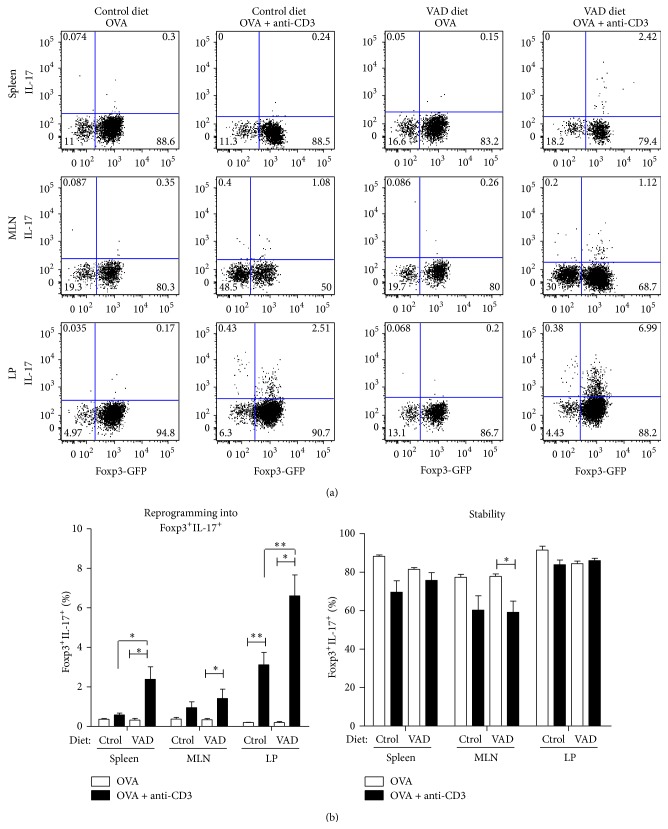
Vitamin A impairs the conversion of iTreg cells into IL-17-producing cells during intestinal inflammation. Highly purified CD45.1^+^ Foxp3^+^ cells generated* in vitro* were transferred into congenic mice (CD45.2^+^) fed a vitamin A-deficient diet (VAD) or control diet. At days 1 and 3 after the adoptive transfer, the recipient mice were injected intraperitoneally with OVA + anti-CD3 to induce inflammation or OVA alone as a control. The lymphoid organs and lamina propria were dissected at day 6, and the cells were analyzed to assess the expression of Foxp3 and IL-17 production on the transferred CD4^+^ cells (CD45.1^+^). (a) Representative graph showing the percentage of Foxp3-GFP expression against IL-17 on the transferred CD4^+^ cells (CD45.1^+^). (b) Quantification of Foxp3^+^IL-17^+^ (left) and Foxp3^+^IL-17^−^ (right) cells for each condition. Means ± SEM are shown. Mann-Whitney *U* test ^*∗*^ *p* < 0.05, ^*∗∗*^ *p* < 0.01, and ^*∗∗∗*^ *p* < 0.001.

We and others have demonstrated that RA favors the generation of Treg cells both* in vivo* and* in vitro* [16, 27]. Moreover, given recent reports demonstrating that Treg cells can convert into the inflammatory Th17 phenotype [24, 25], we decided to investigate the stability and reprogramming of* in vitro*-generated regulatory T (iTreg) cells into IL-17-producing cells during inflammation in the gut. For this experiment, purified Foxp3-GFP^+^ OVA-specific iTreg cells that were generated* in vitro* from CD45.1^+^ mice were transferred into congenic CD45.2^+^ mice. At days 1 and 3 after the adoptive transfer, the recipient mice were intraperitoneally injected with anti-CD3 plus OVA to induce inflammation or with OVA alone as a control (see Supplementary Figure 1 in Supplementary Material available online at http://dx.doi.org/10.1155/2015/137893). On day 6, mononuclear cells were obtained from spleens, mesenteric lymph nodes (MLN), and lamina propria (LP) and were analyzed to assess the expression of Foxp3 (GFP^+^) and IL-17 production on the transferred (CD45.1^+^) cells. The results indicate that, during an acute intestinal inflammation, approximately 2% of the Foxp3^+^ cells upregulate IL-17 expression (IL-17^+^GFP^+^), particularly in the LP. This phenomenon was not observed when Foxp3^+^ cells were transferred into mice immunized with OVA alone (Figure 1), which suggests that the inflammatory conditions in the gut favor Tregs conversion into IL-17-producing cells.

Based on recent results of* in vitro* experiments indicating that natural Treg cells treated with RA resist converting into Th17 and other T helper phenotypes [28], we asked whether RA could also have an effect on the stability of iTreg cells* in vivo*. Because RA is produced abundantly in the gut as a metabolite of vitamin A [29], we evaluated iTreg cell stability and reprogramming in mice fed a vitamin A-deficient diet (VAD). Our results indicate that, under inflammatory conditions, a higher percentage of the adoptively transferred iTreg cells are reprogrammed into IL-17-producing T cells in VAD mice compared with the mice fed a control diet containing RA, particularly in the LP (Figure 1). This finding suggests that RA impairs the reprogramming of Tregs into IL-17-producing cells during inflammation in the gut.

### 3.2. Regulatory T Cells Generated under Different Polarizing Conditions Differentially Express Treg Markers but Present Similar Suppressive Capacity

Based on these results, which indicate a role of RA in Treg stability, and considering the reported role of IL-2 on the development, expansion, and function of Tregs [30], we wondered whether RA and IL-2 affect either the expression of suppressive markers characteristic of Treg cells or the suppressive capacity of Treg cells. To answer this question, we generated Treg cells under different Treg polarizing conditions (with and without RA) and evaluated the expression of several functional markers (ectonucleotidases CD39 and CD73, CTLA4, and LAG3) and the suppressive potential of the generated Treg cells.

In these experiments, naive (CD4^+^CD62L^+^CD44^int^CD25^−^Foxp3^−^) T cells isolated from OTII/Foxp3-GFP reporter mice were activated in the presence of TGF-*β* under three different settings: (a) plus IL-2 (TIL-2 Treg), (b) plus RA (TRA Treg), or (c) plus both RA and IL-2 (TILRA Treg). Under these conditions and as reported by others [16] the presence of RA increased iTreg generation from a low of 4% to a high of 80%, as assessed by Foxp3 expression (Supplementary Figure 2).

**Figure 2:**
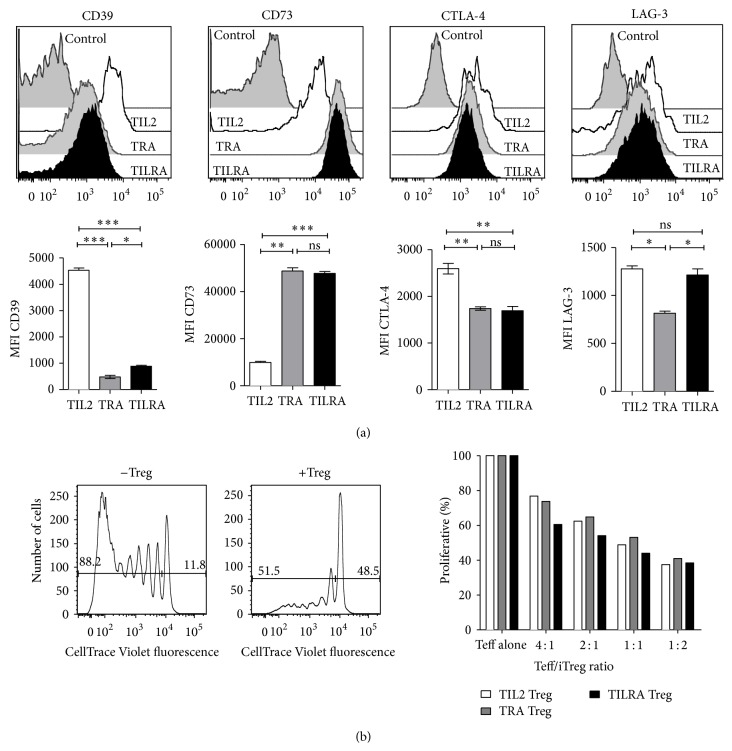
Regulatory T cells generated under different polarizing conditions differentially express Treg markers but present similar suppressive capacity. FACS-sorted naive T cells from the OTII/Foxp3-GFP reporter mice were cultured with splenic APC and anti-CD3 in the presence of TGF-*β* plus IL-2 (TIL-2 Treg), plus RA (TRA Treg), or plus both RA and IL-2 (TILRA Treg). (a) Surface phenotype and Foxp3-GFP expression was analyzed by flow cytometry after 6 days of culture. Representative histograms of CD39, CD73, CTLA4, and LAG3 expression on Foxp3^+^ cells (upper graph). Average MFI ± SEM was derived from three independent experiments (lower graph). ^*∗*^ *p* < 0.05, ^*∗∗*^ *p* < 0.01, and ^*∗∗∗*^ *p* < 0.001. (b) Foxp3-GFP^+^ iTreg cells were sorted and cocultured with CellTrace Violet-labeled naive T cells at different effector , Treg ratios, and then stimulated with anti-CD3 plus CD11c^+^ DCs. Representative histograms of T effector cells proliferation (left). The graph represents the percentage of proliferation of CellTrace Violet-labeled Teff cells (right). Data are representative of three independent experiments.

Regarding the expression of Treg markers, the results presented in Figure 2(a) show that iTreg cells that were generated in the presence of IL-2 but in the absence of RA (TIL-2) presented a higher expression of the CD39 ecto-5′-nucleotidase and CTLA-4 compared with the expression observed in Treg cells that were generated in the presence of RA (TRA and TILRA Treg cells). Moreover, Treg cells generated in the presence of RA exhibited a significantly higher expression of the ecto-5′-nucleotidase CD73 compared with TIL2 Treg cells. Additionally, we detected a significantly lower expression of LAG-3 in TRA Treg compared with that observed in Treg cells that were generated in the presence of IL-2 (TIL2 and TILRA). Taken together, these data suggest that RA decreases the expression of CTLA4 and CD39 while simultaneously increasing CD73 expression. Moreover, IL-2 increases the expression of LAG3.

Next, we compared the suppressive activity of the iTregs that were generated under different polarizing conditions. The Foxp3-GFP^+^ Treg cells generated under all three settings were sorted and cocultured with CellTrace Violet-labeled naive T cells, followed by stimulation with CD11c^+^ DCs and anti-CD3. The results showed that, regardless of the conditions under which the Tregs were generated, they suppressed the effector T cell proliferation to a similar level (Figure 2(b)); this finding indicated that although RA impairs Tregs plasticity, it does not enhance their suppressive capabilities.

### 3.3. IL-2 Confers iTreg Cells Resistance to Th17 Conversion, While RA with IL-2 Sustains Foxp3 Expression

**Figure 3:**
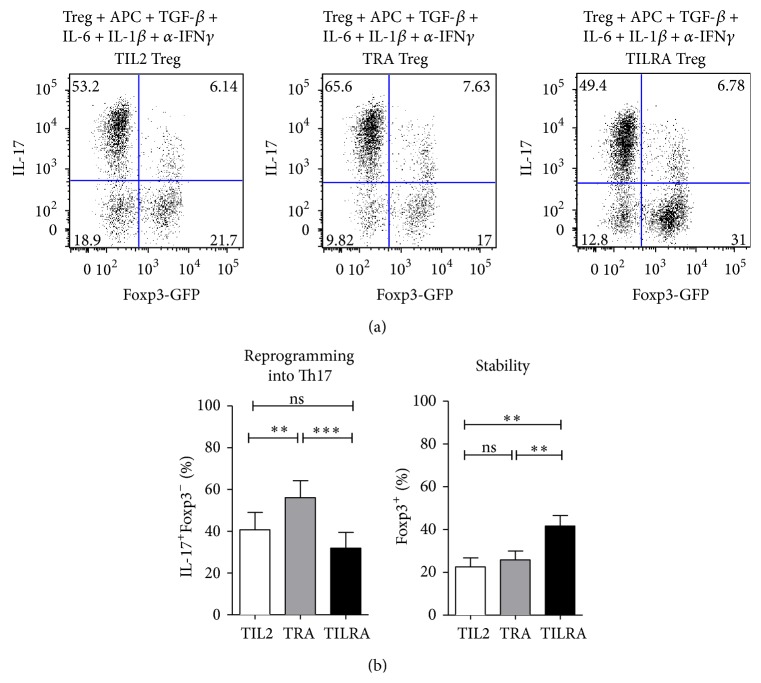
IL-2 confers iTreg resistance to Th17 conversion, while RA and IL-2 cooperate to sustain Foxp3 expression following stimulation under Th17-polarizing conditions. FACS-sorted antigen-specific iTreg cells generated in the presence of TGF-*β* plus IL-2 (TIL-2 Treg), plus RA (TRA Treg), or plus both RA and IL-2 (TILRA Treg) were recultured under Th17-polarizing conditions with IL-6, TGF-*β*1, IL-1*β*, and *α*-IFN*γ*. Six days later, the cells were collected for intracellular IL-17 and Foxp3-GFP staining. (a) Representative staining showing the percentage of Foxp3-GFP expression versus IL-17 content. (b) Values indicate means ± SEM of four separate experiments. Repeated measure ANOVA with Bonferroni's post test ^*∗*^ *p* < 0.05, ^*∗∗*^ *p* < 0.01, and ^*∗∗∗*^ *p* < 0.001.

Based on reports that established that Foxp3 expression is a key factor for the generation and function of Treg cells [31], we decided to investigate whether RA and IL-2 affect the stability of Foxp3. To address this aim, iTreg cells generated under the different conditions were sorted and restimulated for 6 days under Th17-polarizing conditions. The cells were then analyzed by flow cytometry to assess the expression of Foxp-3 and IL-17 production. We observed that when Treg cells that were generated in the presence of both IL-2 and RA (TILRA) were placed under Th17-polarizing conditions, a higher number of cells retained Foxp3^+^ expression (42.0 ± 10.9%) compared with Tregs generated in the presence of IL-2 alone (22.5 ± 9.0%) or RA alone (26.0 ± 9.1%) (Figure 3). Furthermore, the results show that, in the absence of IL-2 (TRA Treg), Treg cells convert to a Th17 phenotype (56.1 ± 18.0%) at a higher frequency compared with TILRA (31.0 ± 16.0%) or TIL-2 (48.0 ± 18.0%) Treg cells. Taken together, these data suggest that the simultaneous presence of RA and IL-2 contributed to a more stable Foxp3 expression, which may favor Treg stability under inflammatory conditions.

## 4. Discussion

Treg cells are involved in maintaining peripheral tolerance and controlling the immune response. Thus, the stability and plasticity of Treg cells might affect their ability to control inflammation. Recently, the imbalanced development and function of IL-17-producing Th17 and Foxp3^+^ Treg cells have been demonstrated to play an important role in autoimmune diseases, including IBD [4, 5]. Therefore, defining the mechanisms and factors that control Treg cell stability/plasticity may contribute to our understanding of the mechanisms involved in the immune deregulation observed in chronic inflammation and autoimmunity and to the design of regulatory T cell-based therapies.

The results of our* in vivo* studies demonstrate that, following adoptive transfer, iTreg cells upregulate IL-17 expression during intestinal inflammation. Previous studies have shown an increase in the presence of IL-17^+^Foxp3^+^CD4^+^ T cells in patients with IBD, which suggests that these double-positive cells may have a dual proinflammatory and regulatory function [32]. In this regard, our results suggest that the IL-17^+^ Foxp3^+^ cells observed in IBD might have been generated from single-positive Foxp3 Treg cells under inflammatory conditions and that Treg cells may contribute to the pathogenesis of gut inflammation via this mechanism.

The importance of vitamin A in the regulation of intestinal immunity has long been recognized. Several published studies in this area have demonstrated that RA downregulates inflammatory responses in murine models of colitis [4, 33] and that vitamin A deficiency can lead to exacerbated experimental colitis [34]. Thus, to study the effect of RA in Treg plasticity* in vivo*, we adoptively transferred iTreg cells to mice that were fed a vitamin A-deficient diet. Interestingly, we observed that vitamin A deficiency favors the reprogramming of Treg cells into IL-17-producing cells during intestinal inflammation. In this context, Xiao and colleagues demonstrated that RA treatment decreased Ag-specific Th17 development by inhibiting IL-16 and IL-23 receptor expression [19]. Moreover, Zhou and colleagues showed that natural Tregs treated with RA were resistant to Th17 conversion due to the decrease in IL-6-R [28]. The importance of the local presence of RA in maintaining a healthy immune response in the gut is reinforced by our results, which indicate that even when Tregs were generated in the presence of RA* in vitro*, vitamin A deficiency favors the reprogramming of iTregs into IL-17-producing cells during intestinal inflammation. Thus, we can speculate that vitamin A deficiency may favor the expression of IL-16 and IL-23 receptors in adoptively transferred iTreg cells during intestinal inflammation in the gut.

Because of the interest in potential therapeutic approaches that utilize the transfer of regulatory T cells, we have explored the role of IL-2 and RA, two factors that are involved in the generation of Treg cells, in their phenotype, function, and stability.

One of the suppressive mechanisms attributed to Treg cells is metabolic disruption through the expression of CD39 and CD73. Although these ectonucleotidases act in concert to convert ATP into immunosuppressive adenosine [35] their cellular expression is dissimilar. Whereas CD39 is expressed primarily on the surface of activated T cells, CD73 is expressed on Foxp3 regulatory T cells and on precursor T cells [36]. Our results show a disparate expression of these enzymes. Thus, adding RA during the* in vitro* differentiation of iTreg cells decreases the expression of CD39 while simultaneously increasing CD73 on the cell surface. Another suppressive mechanism of Treg cells is the control of DC maturation and activation by CTLA-4 and LAG-3 [37, 38]. As shown in Figure 2, RA decreased the expression of CTLA4, whereas IL-2 increased the expression of LAG3. Finally, Treg cells generated under the different polarizing conditions suppressed effector T cell proliferation to a similar level.

Our* in vitro* experiments demonstrate that the generation of iTreg cells in the presence of IL-2 decreases their reprogramming into Th17 cells when stimulated under Th17-polarizing conditions. A previous report showed that IL-2 downregulates IL-6 receptor expression and IL-6 signaling in Treg cells [39]. This downregulation may explain the differences observed in the reprogramming capability between iTregs generated in presence and absence of IL-2. Additionally, we observed that iTreg cells generated in the presence of both RA and IL-2 (TILRA Treg) presented a more stable phenotype than did iTreg generated with either molecule alone. Chen and colleagues suggested the importance of IL-2 in Treg stability by demonstrating that administering IL-2* in vivo* results in the stabilization of Foxp3 expression [40]. Our results suggest that IL-2 and RA might act synergistically to sustain Foxp3 expression. Therefore, the use of both factors may be necessary in the generation of Treg cells for Treg cell-based therapies.

## 5. Conclusions

In conclusion, our data demonstrate that IL-2 and RA not only regulate the level of regulatory T cell production but also determine their phenotype and plasticity. We demonstrated that the presence of IL-2 during the* in vitro* generation of iTreg cells confers resistance to Th17 conversion and that IL-2 cooperates with RA to maintain Foxp3 expression following stimulation under Th17-polarizing conditions. In addition, we showed that local inflammation and vitamin A deficiency favor the reprogramming of Treg cells into IL-17-producing cells. Thus, Tregs stability and plasticity are regulated not only by the signals they receive during generation but also by the signals present in the local microenvironment where they exert their function.

## Supplementary Material

Supplemental information includes two figures. Supplementary Figure 1 shows changes in body weight in anti-CD3 antibody-treated mice and Supplementary Figure 2 includes a flow cytometric analysis of FoxP3 expression during the generation of antigen specific iTreg cells under different po-larizing conditions.
