# Supplementary material for: Vitamin A Impairs the Reprogramming of Tregs into IL-17-Producing Cells during Intestinal Inflammation
Source: Biomed Res Int. 2015 Oct 25;2015:137893. doi: 10.1155/2015/137893 (PMC4637025; doi:10.1155/2015/137893)
Supplement: Supplementary file 1 — Supplemental information includes two figures. Supplementary Figure 1 shows changes in body weight in anti-CD3 antibody-treated mice and Supplementary Figure 2 includes a flow cytometric analysis of FoxP3 expression during the generation of antigen specific iTreg cells under different po-larizing conditions. [file 137893.f1.pdf]

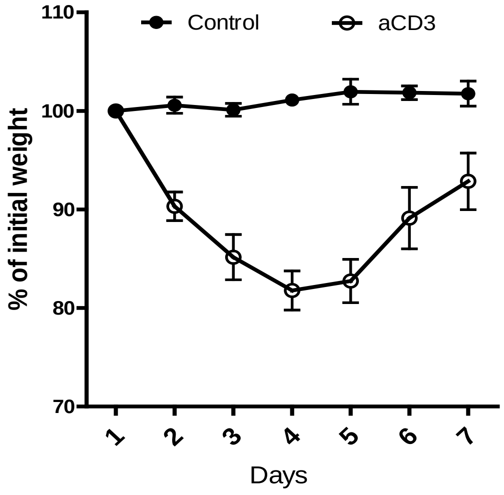

Supplementary figure 1: Intestinal inflammation as determined by changes in body weight after anti-CD3 antibody treatment. Mice were injected intraperitoneally two times (day 1 and 3) with either anti-CD3 antibody or PBS as a control. Body weight was recorded daily from day 1 to 7. The changes in body weight is expressed as a percentage of the initial weight at day 1. Mean ± SEM are shown.

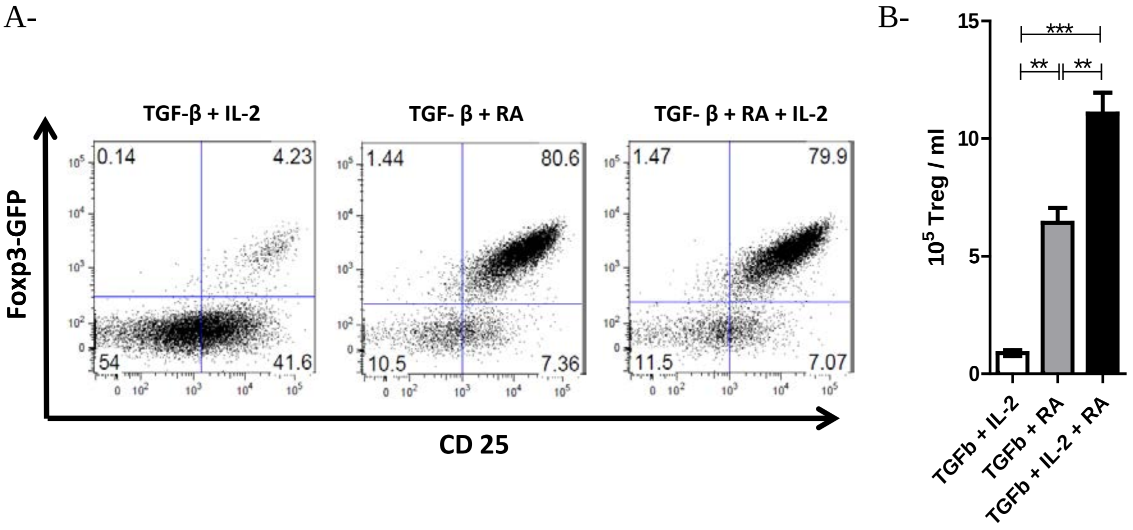

A-
TGF-β + IL-2
TGF- β + RA
TGF- β + RA + IL-2
0.14
4.23
54
41.6
1.44
80.6
10.5
7.36
1.47
79.9
11.5
7.07
Foxp3-GFP
CD 25
B-
***
**
**
10^5 Treg / ml
15
10
5
0
TGFb + IL-2
TGFb + RA
TGFb + IL-2 + RA
